# Screen Positivity and Feasibility of Newborn Thyroid Screening Using Postnatal Venous Thyroid-Stimulating Hormone: A Retrospective Cohort Study From Northeast India

**DOI:** 10.7759/cureus.114332

**Published:** 2026-08-11

**Authors:** Mampy Das, Rosina Ksoo, Sukalyan Halder, Himesh Barman, Polina Boruah

**Affiliations:** 1 Department of Pediatrics, North Eastern Indira Gandhi Regional Institute of Health and Medical Sciences (NEIGRIHMS), Shillong, IND; 2 Department of Obstetrics and Gynecology, North Eastern Indira Gandhi Regional Institute of Health and Medical Sciences (NEIGRIHMS), Shillong, IND; 3 Department of Biochemistry, North Eastern Indira Gandhi Regional Institute of Health and Medical Sciences (NEIGRIHMS), Shillong, IND

**Keywords:** congenital hypothyroidism, neonatal screening, newborn infant, thyroid function tests, thyrotropin

## Abstract

Introduction: Congenital hypothyroidism (CH) is a preventable cause of intellectual disability when detected and treated early. Postnatal thyroid-stimulating hormone (TSH)-based newborn screening is recommended in India, but evidence from resource-limited settings remains scarce. This study aimed to determine the screen positivity rate and evaluate the feasibility of postnatal venous TSH-based screening.

Methods: This retrospective study included live births at a tertiary care hospital in Northeast India between March 2023 and November 2024 who underwent CH screening using venous TSH estimation at 48-72 hours of life. A TSH value of >20 mIU/L was considered screen-positive according to the Indian Society for Pediatric and Adolescent Endocrinology guidelines. Screen positivity rates were calculated, and associations with clinical variables were explored. Continuous variables were summarized using descriptive statistics, and associations were evaluated using the chi-square test or Fisher's exact test, as appropriate. A p value of <0.05 was considered statistically significant.

Results: Of 1,622 live births, 1,132 (69.8%) underwent screening, of whom 1,064 (94.0% of those screened) had analyzable results. Most exclusions resulted from early discharge before sampling and inadequate blood samples. The mean (standard deviation) TSH value was 4.49 (5.19) mIU/L. Nine newborns were screen-positive, yielding a screen positivity rate of 0.85% (8.5 per 1,000 newborns), which decreased to 0.47% after excluding clinically unwell neonates. Among screen-positive newborns, median TSH values were lower in preterm than in term newborns (25.44 vs. 37.52 mIU/L). Maternal thyroid disorder, present in 22.4% of mothers, was not significantly associated with abnormal neonatal TSH (p = 0.73).

Conclusion: Postnatal venous TSH-based screening was feasible but associated with moderate attrition. The observed screen positivity reinforces the importance of universal newborn screening for CH in this region. Strengthening follow-up and optimizing sample collection, including evaluation of cord blood screening, may enhance the effectiveness of newborn screening programs.

## Introduction

Congenital hypothyroidism (CH), if left untreated, can result in severe intellectual disability and impaired growth [1-3]. Early initiation of L-thyroxine replacement is the simplest and most effective strategy for preventing these adverse outcomes [1-4]. The Indian Council of Medical Research Task Force study (2007-2012), conducted across four metropolitan cities in India, reported an incidence of CH of approximately one in 1,130 live births [5]. The incidence of CH in India has been reported to be higher than the global average, with estimates ranging from one in 1,130 to one in 2,804 live births, compared with a worldwide incidence of one in 3,000-4,000 live births [5,6]. Newborn screening for CH has therefore been recognized as a public health priority [3,5,6].

The Indian Society for Pediatric and Adolescent Endocrinology (ISPAE) recommends screening using thyroid-stimulating hormone (TSH) estimation, either from cord blood or postnatal samples collected at 48-72 hours of life [5,7,8]. In a meta-analysis, Anne and Rahiman reported that postnatal samples were associated with lower screen positivity rates than cord blood; however, cord blood screening demonstrated better compliance with confirmatory testing [9].

However, published data on the incidence of CH and the performance of newborn screening programs from resource-limited and geographically distinct regions, such as Northeast India, remain limited. In addition, information on screening coverage and program completion remains sparse in such settings. Since March 2023, newborn screening for CH using postnatal venous samples has been implemented at our tertiary care center in Northeast India. Therefore, this retrospective cohort study aimed to determine the screen positivity rate and evaluate the feasibility of this screening approach in our setting.

## Materials and methods

Study design and setting

This retrospective cohort study was conducted in the Department of Pediatrics at a tertiary care hospital between March 2023 and November 2024. Ethical approval was obtained from the Institutional Ethics Committee.

Study population

During the study period, 1,622 live births were identified through a consecutive sampling approach. Of these, 1,132 newborns underwent screening for CH using postnatal venous TSH estimation, and 1,064 had results available for analysis. As per our institutional protocol, 1 mL of venous blood was collected between 48 and 72 hours of life and analyzed for serum TSH (mIU/L) using a chemiluminescence immunoassay on a Beckman Coulter DxI 800 analyzer (Beckman Coulter, Inc., Brea, CA). Reports were recorded in a secure offline registry. Infants with missing reports were not excluded a priori; rather, missing data resulted from early discharge or inadequate sample collection requiring repeat testing. A TSH value of >20 mIU/L was considered screen-positive according to ISPAE recommendations [4].

Relevant clinical data for screen-positive newborns were extracted using a predefined data collection format and included maternal thyroid status and neonatal characteristics (gestational age, sex, birth weight, comorbidities, and TSH value). No routine confirmatory testing data were available; therefore, the study outcome was screen positivity rather than confirmed CH.

Statistical analysis

Data were entered into Microsoft Excel (Microsoft Corporation, Redmond, WA) and analyzed using Epi Info™ version 3.5.4 (Centers for Disease Control and Prevention, Atlanta, GA, USA). Categorical variables were expressed as frequencies and percentages; continuous variables were expressed as mean (standard deviation (SD)) or median (interquartile range (IQR)), as appropriate. The screen positivity rate was calculated as the proportion of newborns with TSH values >20 mIU/L and expressed as both a percentage and per 1,000 screened newborns. Associations between categorical variables were evaluated using the chi-square test or Fisher's exact test, as appropriate, according to the expected cell counts. A p value of <0.05 was considered statistically significant.

## Results

**Figure 1:**
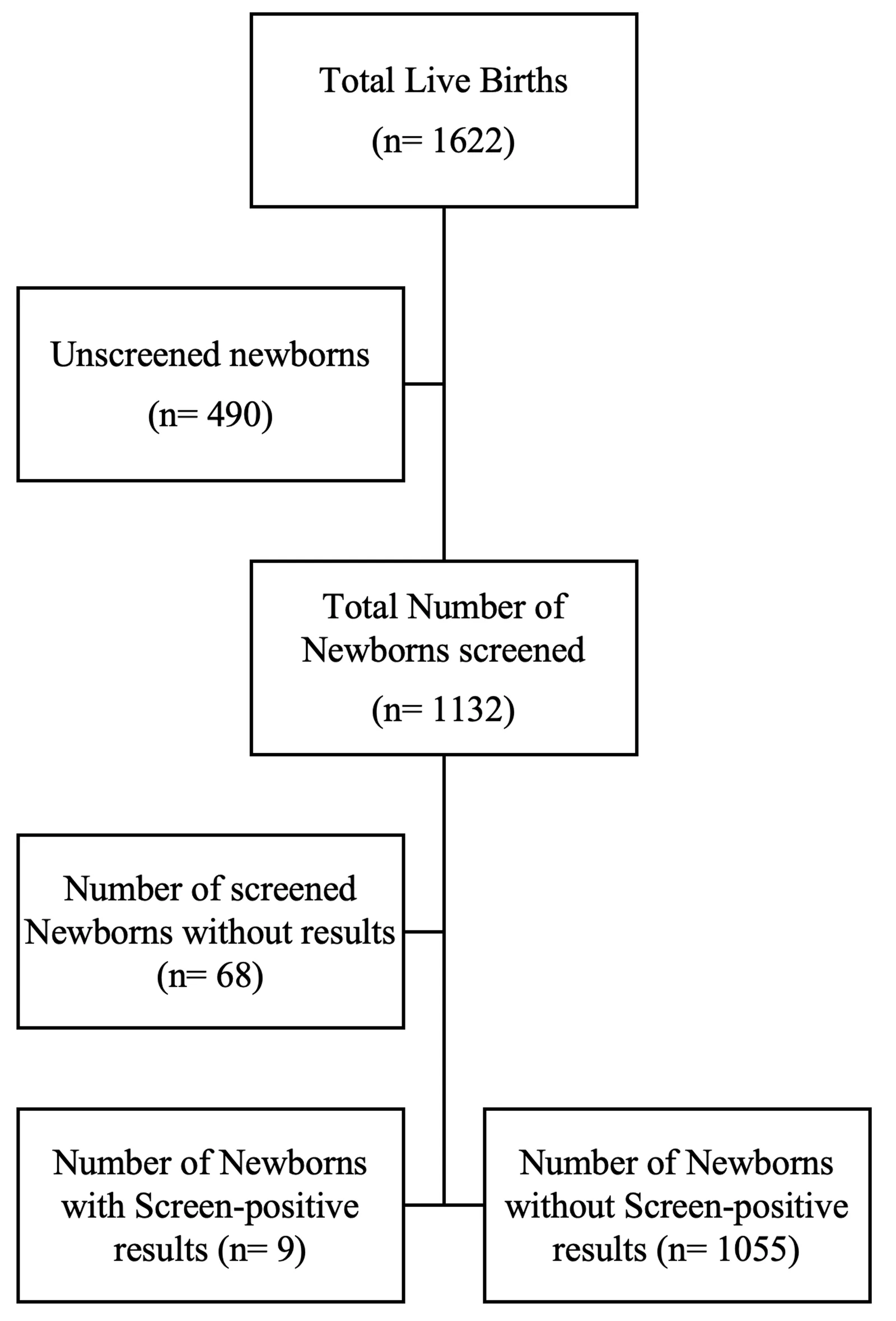
Flow diagram of newborn thyroid screening outcomes

A total of 1,622 live births were recorded during the study period, of which 1,132 newborns underwent thyroid screening (Figure 1).

The screened cohort (n = 1,132) comprised 519 male and 613 female newborns, corresponding to a male-to-female ratio of 0.85:1. TSH results were available for analysis in 1,064 newborns, representing 94.0% of screened newborns and 65.6% of all live births. The mean (SD) venous TSH among all screened newborns was 4.49 (5.19) mIU/L. Nine newborns were identified as screen-positive (TSH >20 mIU/L), yielding a screen positivity rate of 0.85% (8.5 per 1,000 newborns; approximately one in 118) (Table 1).

**Table 1:** Distribution of venous TSH values among screened newborns according to ISPAE screening categories TSH: thyroid-stimulating hormone; ISPAE: Indian Society for Pediatric and Adolescent Endocrinology

TSH level (mIU/L)	Newborns, n (%)
<20	1,055 (99.16)
20-40	6 (0.56)
40-80	2 (0.19)
>100	1 (0.09)
Total	1,064 (100)

The characteristics of the screen-positive newborns are summarized in Table 2.

**Table 2:** Characteristics of screen-positive newborns (n = 9) TSH: thyroid-stimulating hormone; SD: standard deviation

Characteristic	Value
Sex, n (%)	
Male	4 (44.4%)
Female	5 (55.6%)
Preterm birth, n (%)	3 (33.3%)
Low birth weight, n (%)	4 (44.4%)
Mean TSH value, mIU/L (SD)	40.7 (26.0)
TSH >40 mIU/L, n (%)	2 (22.2%)
Comorbidities, n (%)	
Hypoglycemia	1 (11.1%)
Neonatal hyperbilirubinemia	1 (11.1%)
Birth asphyxia	2 (22.2%)
Respiratory distress	2 (22.2%)

Among these, four newborns were clinically unwell at the time of sampling. After excluding these newborns, the screen positivity rate was 0.47% (5/1,064), equivalent to 4.7 per 1,000 screened newborns (approximately one in 213). Among screen-positive newborns, the median (IQR) TSH value was 25.44 mIU/L (23.22-43.32 mIU/L) in preterm newborns and 37.52 mIU/L (29.66-40.76 mIU/L) in term newborns. Maternal thyroid disorder was present in 238 (22.4%) mothers (Table 3).

**Table 3:** Association between maternal thyroid disorder and neonatal screen positivity Fisher’s exact test, p = 0.73 TSH: thyroid-stimulating hormone

Maternal thyroid disorder	Screen-positive (TSH >20 mIU/L) in baby	Screen-negative TSH <20 mIU/L in baby	Total
Yes	1	237	238
No	8	818	826
Total	9	1,055	1,064

Fisher's exact test showed no significant association between maternal thyroid disorder and neonatal screen positivity (p = 0.73).

## Discussion

The screen positivity rate of 0.85% (8.5 per 1,000 screened newborns) observed in our study exceeded the reported incidence of CH in India and globally [5]. This difference is expected because screen positivity reflects screening performance rather than the true incidence of confirmed CH, and screening performance is influenced by assay methodology, specimen type, timing of sample collection, and TSH cutoff values [8,10]. Using a TSH cutoff of >20 mIU/L as per ISPAE guidelines for postnatal venous samples, this rate is higher than that reported in the meta-analysis by Anne and Rahiman (0.19%) for postnatal screening, although lower rates have consistently been reported with this method than with cord blood screening (5.6%) [4,9]. Notably, after excluding clinically unwell newborns, the screen positivity rate decreased to 0.47% (4.7 per 1,000 screened newborns), suggesting a possible contribution of illness-related transient TSH elevation.

The mean (SD) TSH value (4.49 (5.19) mIU/L) observed in our cohort was lower than that reported by Singh et al. from the neighboring state of Manipur (8.83 (7.06) mIU/L), but comparable to that reported by Gaur et al., who found a mean (SD) cord blood TSH value of 7.0 (5.3) mIU/L, using cord blood screening [11,12]. In contrast, Al Juraibah et al. reported substantially higher mean TSH values (29.8 mIU/L) using heel-prick sampling processed by mass spectrometry [13]. These variations likely reflect differences in sampling timing, specimen type (cord blood, postnatal venous samples, or dried blood spots), and differences in screening thresholds used across newborn screening programs [7,8,10,14]. Therefore, direct comparisons should be interpreted with caution.

Gaur et al. reported higher mean (SD) TSH values in extremely preterm newborns [12]. Similarly, Anne and Rahiman reported a higher prevalence of CH among preterm neonates [9]. In contrast, median TSH values in our study were lower in preterm than in term newborns; however, given the small sample size (n = 9) and overlapping IQRs, this finding should be interpreted cautiously. Preterm and sick newborns are known to have an increased risk of abnormal TSH levels, including both false-positive and false-negative results [3,4,7,15]. Several perinatal factors, including gestational age, birth weight, and sex, have been shown to influence neonatal TSH values and should be considered when interpreting screening results [14].

Maternal thyroid disorder was not associated with abnormal neonatal TSH (p = 0.73) in our cohort. Although previous studies, including a recent meta-analysis and prospective studies involving infants born to hypothyroid mothers, have reported a higher risk of CH, transient neonatal hyperthyrotropinemia, and an association between maternal and neonatal TSH values, no such association was observed in our study [16-18]. This difference should be interpreted cautiously because our analysis included mothers with both hypothyroidism and hyperthyroidism and evaluated screen positivity rather than confirmed CH. Nevertheless, this finding supports the importance of universal rather than selective screening [8].

Only 65.6% of all live births had TSH results available for analysis, highlighting gaps in the screening pathway. The primary reasons for missing reports included early discharge of the mother-baby dyad before 48 hours of life and inadequate sample collection requiring repeat testing, both of which contributed to loss before analyzable results were obtained. Notably, no maternal refusal for screening was observed, indicating good acceptability of the program. This suggests that the observed gaps are likely related to system-level factors rather than parental acceptance [3]. Al Juraibah et al. and Alameer et al. similarly reported that, despite comparable diagnostic performance, cord blood TSH screening demonstrated better recall rates than heel-prick sampling [13,15]. Similarly, Indian studies have demonstrated that cord blood screening is a practical approach in settings where early postnatal discharge limits timely postnatal sample collection, thereby improving screening completion and follow-up [7,19,20]. In response to these challenges, cord blood sampling has been adopted as a quality-improvement initiative at our center, and its performance has yet to be evaluated. This approach is supported by evidence from large hospital-based newborn screening programs in India demonstrating the feasibility of cord blood TSH screening [7,8]. Alameer et al. and Zwaveling-Soonawala et al. also recommended cord blood TSH screening, especially in resource-limited settings [15,21].

This study is limited by its retrospective design and the lack of detailed follow-up data to confirm the diagnosis of CH. In addition, the large proportion of newborns without analyzable TSH results may have introduced selection bias. Furthermore, because confirmatory testing was not available, the true incidence of CH could not be determined. However, this study provides real-world data on newborn CH screening from a geographically underrepresented region. Further prospective studies incorporating confirmatory testing and long-term outcomes are warranted to better define the true burden of CH in this region.

## Conclusions

Postnatal venous TSH-based newborn screening was feasible in our setting, yielding a screen positivity rate of 0.85%. However, a substantial proportion of newborns did not have analyzable results due to early discharge and inadequate sample collection, highlighting gaps in screening completion. These findings support the need for universal newborn screening for CH in Northeast India and underscore the importance of strengthening follow-up pathways and optimizing sample collection strategies. Evaluation of cord blood TSH screening may further improve screening completion and program effectiveness in resource-limited settings.
